# Kikuchi-Fujimoto Disease in a Patient With Systemic Lupus Erythematosus: A Case Report

**DOI:** 10.7759/cureus.116332

**Published:** 2026-09-15

**Authors:** Amna Sayah Abdulla Alhammadi, Almaha Khaled Almahri, Nithin Narayan, Rabea Ebrahim Shaheen

**Affiliations:** 1 Internal Medicine, Sheikh Shakhbout Medical City, Abu Dhabi, ARE

**Keywords:** hemophagocytic lymphohistiocytosis (hlh), kikuchi-fujimoto disease, kikuchi-fujimoto disease and systemic lupus erythematosus, necrotizing histiocytic lymphadenitis, necrotizing lymphadenitis, systemic lupus erythematosus

## Abstract

Kikuchi-Fujimoto disease (KFD), or histiocytic necrotizing lymphadenitis, is a rare, self-limiting syndrome characterized by fever and lymphadenopathy, most commonly cervical, though other nodal regions may be involved. It is well recognized to occur in patients with systemic lupus erythematosus (SLE), with which it shares substantial clinical and histopathologic overlap, often mimicking a lupus flare or a lymphoproliferative disorder. We report the case of a 43-year-old woman with a known history of SLE, maintained on hydroxychloroquine and low-dose prednisolone, who presented with a two-week history of fever, chills, rigors, and generalized body aches. She was found to have elevated inflammatory markers, hypocomplementemia, a markedly elevated antinuclear antibody titer, and elevated anticardiolipin IgM, with imaging demonstrating marked right axillary and bilateral pelvic/iliac lymphadenopathy. Excisional lymph node biopsy demonstrated extensive necrotizing lymphadenitis with karyorrhectic debris and a predominantly T-cell infiltrate, without granulomas, and with negative infectious and Epstein-Barr virus studies, consistent with KFD. Bone marrow examination excluded an underlying hematologic malignancy, and a low HScore made hemophagocytic lymphohistiocytosis unlikely. The patient was treated with escalation of corticosteroid therapy and continuation of hydroxychloroquine, achieving clinical recovery with resolution of fever. This case highlights the importance of recognizing KFD as a cause of fever and lymphadenopathy in patients with SLE, and underscores the value of histopathologic confirmation in guiding appropriate management.

## Introduction

Kikuchi-Fujimoto disease (KFD), also known as histiocytic necrotizing lymphadenitis, is an uncommon, generally benign, and self-limiting disorder characterized by fever and tender lymphadenopathy, most commonly cervical, though other nodal regions, including axillary and generalized distributions, may be involved [1].

It predominantly affects young women, with a disproportionate representation among East Asian populations, although cases have been reported worldwide [2]. The etiology remains unknown, though an aberrant, self-limited T-cell-mediated immune response to an unidentified antigenic trigger has been proposed [1].

KFD holds particular clinical importance because of its well-documented association with SLE. It may precede an eventual SLE diagnosis, arise concurrently in a patient already carrying the diagnosis, or follow an established diagnosis during a disease flare [2-4]. Because its histologic features can closely resemble lupus lymphadenitis, and its clinical presentation can mimic lymphoma or infection [1,4], KFD is frequently underdiagnosed and requires a high index of suspicion, excisional lymph node biopsy, and careful histopathologic and immunohistochemical evaluation to confirm [1].

We report a case of KFD occurring in a patient with pre-existing SLE, presenting as prolonged fever and marked lymphadenopathy, to highlight the clinical and diagnostic features of this rare condition and the workup required to establish the diagnosis.

## Case presentation

A 43-year-old woman with a known history of SLE, diagnosed in 2017 with a predominantly articular phenotype (joint pain and arthritis), maintained on hydroxychloroquine 300 mg daily and prednisolone 5 mg daily, presented to our hospital with a two-week history of fever associated with chills, rigors, and drenching sweats. She additionally reported generalized body aches without weight loss. A comprehensive review of systems was otherwise unremarkable: she denied chest pain, cough, dyspnea, upper respiratory symptoms, abdominal pain, nausea, vomiting, dysuria, change in urinary frequency or color, joint pain, rash, oral ulcers, or ocular symptoms. There was no family history of malignancy and no recent travel history.

On admission, she was febrile (38.3°C) and tachycardic (104 bpm), with a blood pressure of 105/61 mmHg and a respiratory rate of 18 breaths per minute. On examination, the patient was alert, oriented, and not in acute distress. There was no pallor, jaundice, rash, or peripheral edema. She had cervical and right axillary lymphadenopathy. Cardiovascular and respiratory examinations were unremarkable. The abdomen was soft and non-tender, with no hepatosplenomegaly. Musculoskeletal and neurological examinations were unremarkable.

On evaluation, inflammatory markers were elevated, and hemoglobin was reduced with a microcytic, hypochromic picture; renal function, electrolytes, coagulation parameters, and bilirubin were normal. Laboratory investigations at presentation are detailed in Table 1.

**Table 1:** Laboratory investigations at presentation. eGFR: estimated glomerular filtration rate, CKD-EPI: Chronic Kidney Disease Epidemiology Collaboration, LDH: lactate dehydrogenase, INR: international normalized ratio, APTT: activated partial thromboplastin time, PT: prothrombin time, ESR: erythrocyte sedimentation rate, CRP: C-reactive protein. The arrows indicate deviation from normal (↑ elevated, ↓ reduced, ↑↑/↓↓ markedly abnormal).

Parameter	Normal range	Value at presentation
Hemoglobin (g/L)	120-160	74 ↓↓
White blood cells (×10⁹/L)	4.0-11.0	4.07 (normal)
Platelets (×10⁹/L)	150-400	449 ↑
Serum creatinine (µmol/L)	45-90	52 (normal)
eGFR (CKD-EPI) (mL/min/1.73 m²)	>60	118 (normal)
Sodium (mmol/L)	136-145	137 (normal)
Bilirubin total (µmol/L)	3-21	3.6 (normal)
Bilirubin direct (µmol/L)	0-5	2.9 (normal)
LDH (U/L)	135-225	272 ↑
AST (U/L)	5-40	16 (normal)
ALT (U/L)	5-40	12 (normal)
Fibrinogen (g/L)	2.0-4.0	3.5 (normal)
Triglycerides (mmol/L)	<1.7	1.46 (normal)
INR	0.9-1.1	1.01 (normal)
APTT (seconds)	25-40	36.4 (normal)
PT (seconds)	10-14	13.40 (normal)
Ferritin (µg/L)	13-150	3464 ↑↑
ESR (mm/hr)	0-20	99 ↑↑
CRP (mg/L)	<5	41.4 ↑↑

The peripheral blood smear showed hypochromasia with occasional schistocytes. Chest radiography (Figure 1) and echocardiography were unremarkable.

**Figure 1:**
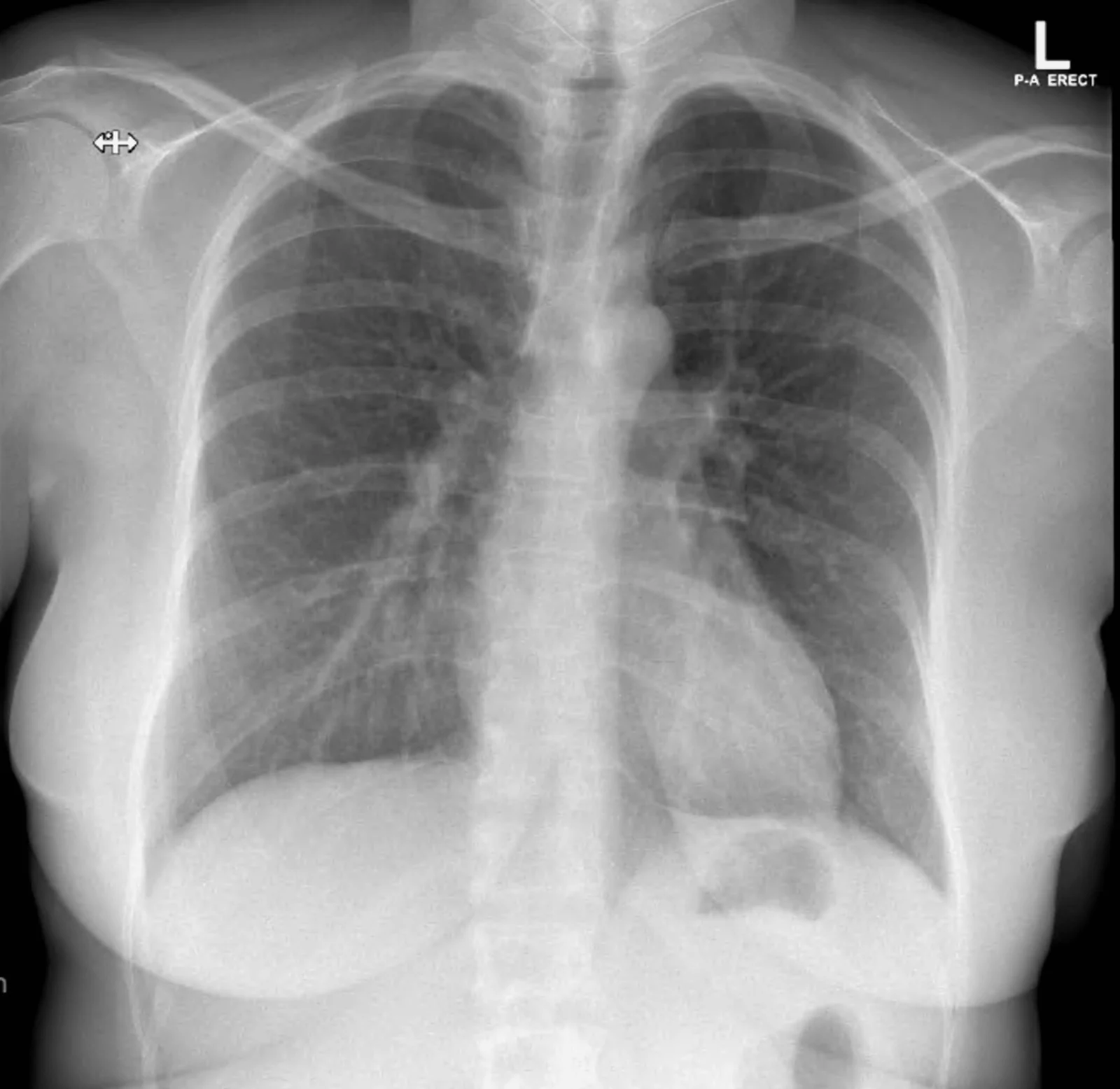
Postero-anterior erect chest radiograph showing no consolidation, pleural effusion, or pneumothorax.

Autoimmune workup revealed a markedly elevated antinuclear antibody titer, low complement levels, and elevated anticardiolipin IgM, with polyclonal hypergammaglobulinemia on serum protein electrophoresis. Screening for HIV, hepatitis B and C, and influenza A and B was negative, and blood and urine cultures were negative. Immunological and serological investigations are shown in Table 2.

**Table 2:** Immunological and serological investigations. ANA: antinuclear antibody, anti-dsDNA: anti-double-stranded DNA antibody, HIV: human immunodeficiency virus.

Parameter	Normal range	Value
ANA titer	<1:80 (negative)	1:1500 ↑↑ (positive)
Anti-dsDNA (IU/mL)	<30 (negative)	361 ↑↑ (positive)
Complement C3 (g/L)	0.9-1.8	0.79 ↓
Complement C4 (g/L)	0.1-0.4	0.12 (low-normal)
Anticardiolipin IgM (IU/mL)	<20 (negative)	361 ↑↑ (positive)
HIV	Negative	Negative
Hepatitis B surface antigen	Negative	Negative
Hepatitis C antibody	Negative	Negative
Influenza A/B (antigen)	Negative	Negative
Blood culture	No growth	No growth
Urine culture	No growth	No growth

Urinalysis showed only trace proteinuria without hematuria, pyuria, or leukocyte esterase or nitrite positivity, with a protein-to-creatinine ratio at the upper limit of normal, arguing against significant active renal involvement at presentation. Urinalysis findings are shown in Table 3.

**Table 3:** Urinalysis findings. HPF: high-power field.

Parameter	Reference	Result
Color	Straw to dark yellow	Dark yellow
Specific gravity	1.005-1.030	1.025
pH	4.5-8.0	6.0
Protein	Negative/trace	Trace
Leukocyte esterase	Negative	Negative
Nitrite	Negative	Negative
Blood	Negative	Negative
White blood cells	Negative	None seen
Red blood cells	Negative	None seen
Protein-to-creatinine ratio (g/g Cr)	<0.2	0.20 (upper-normal)

Contrast-enhanced computed tomography of the chest, abdomen, and pelvis revealed multiple enlarged right axillary lymph nodes, the largest measuring 3.3 × 2.2 cm, without hilar or mediastinal lymphadenopathy (Figure 2).

**Figure 2:**
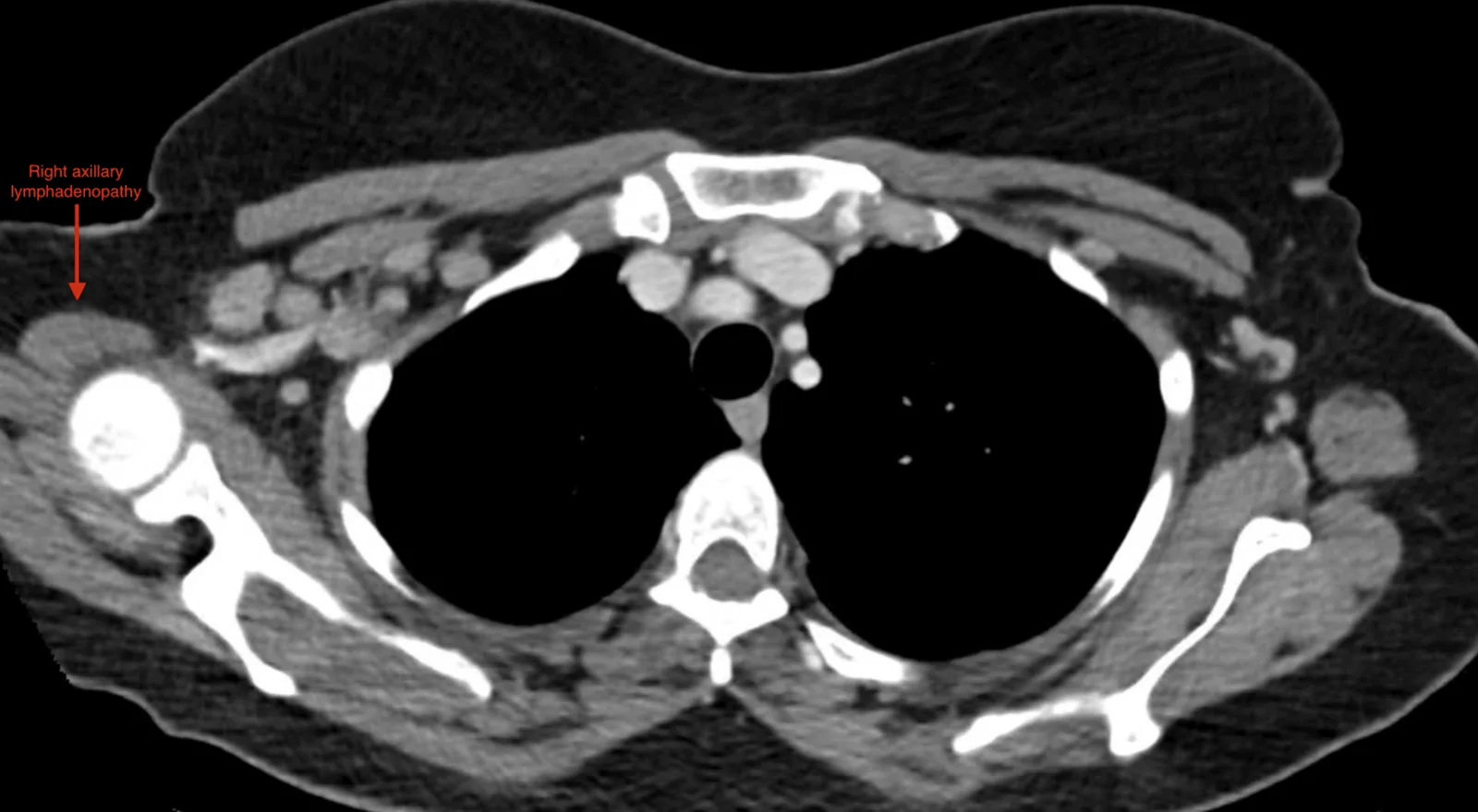
Axial contrast-enhanced CT of the chest at the level of the axillae, showing enlarged right axillary lymph nodes.

Additional enlarged lymph nodes were noted along the bilateral common and external iliac chains and pelvic sidewalls, the largest measuring up to 2.3 × 1.3 cm (Figure 3).

**Figure 3:**
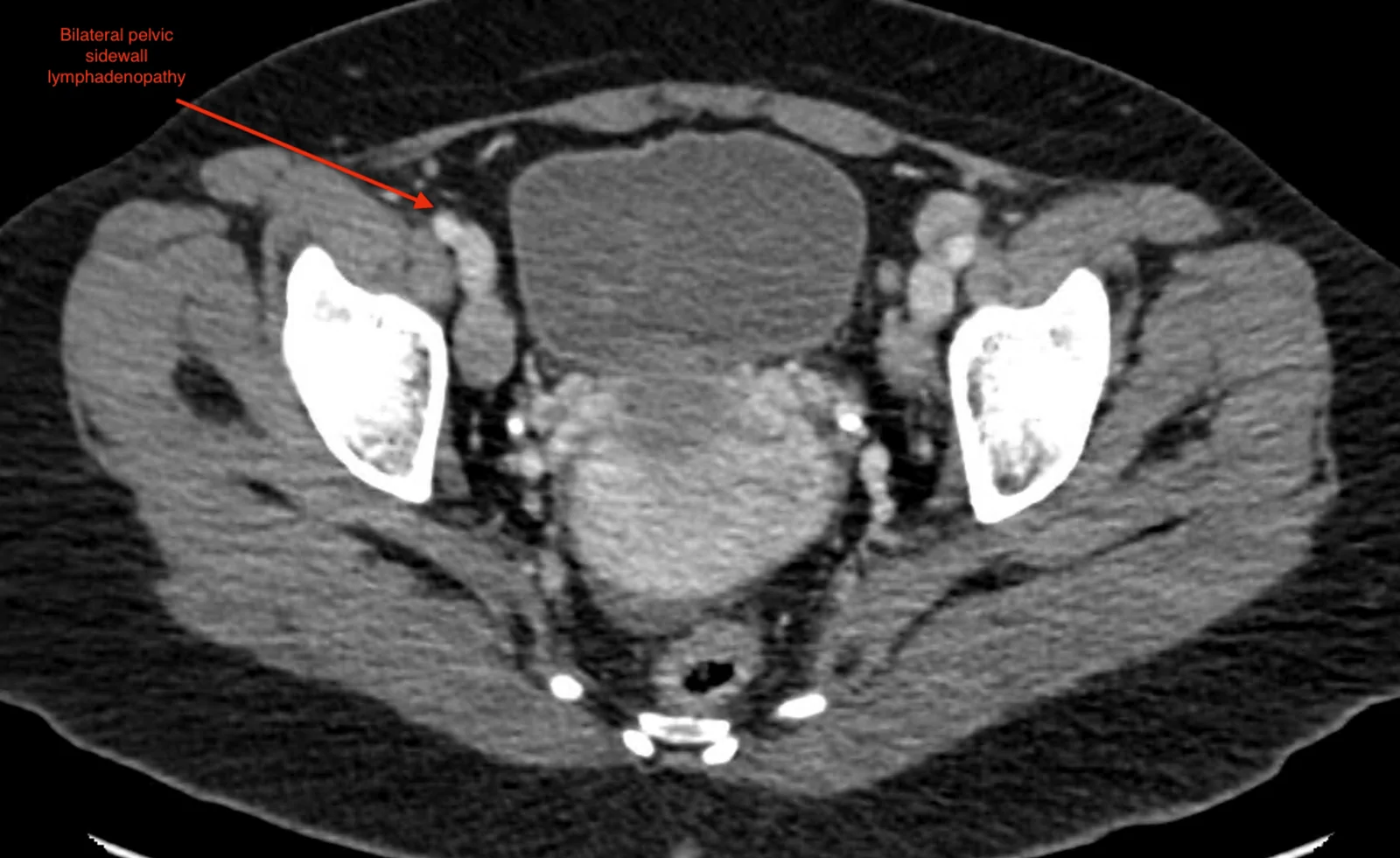
Axial contrast-enhanced CT of the pelvis, showing enlarged bilateral iliac and pelvic sidewall lymph nodes.

Incidental findings included a well-circumscribed 2.2 cm splenic hilar nodule most consistent with an accessory spleen and a partially cystic left ovarian lesion (2.2 × 2.2 cm) without ovarian enlargement. The liver, spleen, and other abdominal organs were unremarkable (Figure 4).

**Figure 4:**
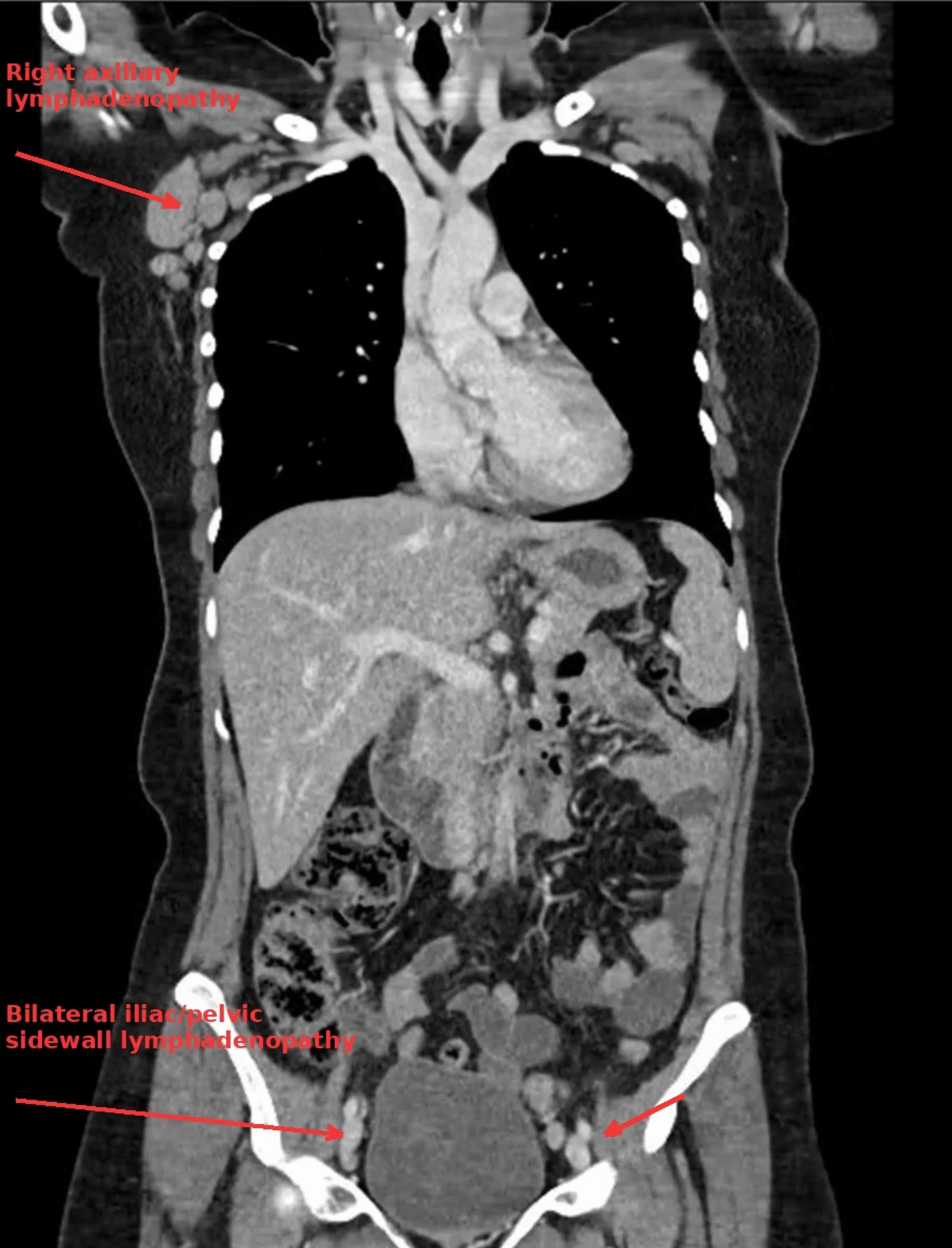
Coronal reconstructed contrast-enhanced CT of the chest, abdomen, and pelvis, showing the overall extent and distribution of lymphadenopathy and unremarkable abdominal organs.

Given the significant lymphadenopathy and concern for a lymphoproliferative disorder, an excisional right axillary lymph node biopsy was performed. Histopathology demonstrated extensive necrotizing lymphadenitis with karyorrhectic debris and apoptotic bodies engulfed by histiocytes, with no granulomas or significant neutrophilic infiltrate. Immunohistochemistry revealed a predominantly T-cell infiltrate (CD3/CD8 positive) with preserved reactive B-cell follicles. EBV, fungal, and acid-fast bacillus studies were negative. Overall, the findings favored KFD, with lupus lymphadenitis and an occult lymphoproliferative process considered less likely. Bone marrow aspiration and biopsy were performed to exclude an underlying hematologic malignancy in view of the significant lymphadenopathy and cytopenias. Flow cytometry showed no increase in blasts (<1%) and no abnormal lymphoid population or immunophenotypic evidence of acute leukemia, supporting the absence of an underlying hematologic malignancy.

Hematology was consulted for progressive anemia with a declining, low-normal white blood cell count, while the platelet count remained preserved and, in fact, mildly elevated. Peripheral blood smear showed hypochromasia with occasional schistocytes. During admission, ferritin levels increased markedly (Table 1). Given the presence of fever, anemia, hyperferritinemia, and lymphadenopathy in the setting of SLE, secondary hemophagocytic lymphohistiocytosis (HLH) was considered, and repeat evaluation for HLH/macrophage activation syndrome and autoimmune hemolysis was completed. Current findings did not support HLH, with an HScore of 92 corresponding to an estimated probability of approximately 1%. Ferritin was elevated at 3464 µg/L, and LDH was mildly elevated at 272 IU/L; however, triglycerides (1.46 mmol/L) and haptoglobin were within acceptable limits, arguing against active HLH or significant ongoing hemolysis.

A diagnosis of KFD in the setting of serologically active SLE was established based on the clinical presentation and lymph node histopathology. Given the progressive anemia with a low-normal leukocyte count, biopsy-confirmed necrotizing lymphadenitis, and suspected immune-mediated cytopenia in the setting of serologically active SLE, corticosteroid therapy was intensified. Her home prednisolone dose of 5 mg daily was increased to prednisone 1 mg/kg/day (60 mg orally once daily), while hydroxychloroquine 300 mg daily was continued. Following escalation of immunosuppressive therapy, the patient demonstrated significant clinical improvement, with resolution of fever and stable vital signs. She was discharged afebrile and clinically stable on prednisone 60 mg daily and hydroxychloroquine 300 mg daily, with planned outpatient reassessment and corticosteroid dose adjustment. At subsequent follow-up with rheumatology and hematology, she continued to demonstrate clinical improvement, with sustained resolution of fever and improvement in her overall symptoms, allowing for adjustment of the corticosteroid dose according to her clinical response.

## Discussion

The differential diagnosis of fever and lymphadenopathy in patients with SLE is broad and includes lupus lymphadenitis, lymphoma, infectious lymphadenitis, hemophagocytic lymphohistiocytosis (HLH), and KFD. KFD is a rare, generally self-limiting necrotizing lymphadenitis with a well-recognized association with SLE and may precede, occur concurrently with, or follow SLE diagnosis [1,3]. Diagnosis relies primarily on lymph node histopathology, which typically demonstrates necrosis with abundant karyorrhectic debris, histiocytic infiltration, and a predominantly T-cell infiltrate with a relative paucity of neutrophils [1].

The disease usually manifests with cervical lymphadenopathy, often accompanied by fever and constitutional symptoms such as fatigue, night sweats, weight loss, headache, and myalgia [5]. In our patient, however, lymphadenopathy predominantly involved the right axillary and bilateral iliac and pelvic regions, representing an atypical distribution that raised initial concern for lymphoma. KFD is a recognized mimic of lymphoma, emphasizing the importance of tissue diagnosis in atypical presentations [4]. In our patient, excisional lymph node biopsy demonstrated extensive necrotizing lymphadenitis with karyorrhectic debris and apoptotic bodies engulfed by histiocytes, with no granulomas or significant neutrophilic infiltrate. Immunohistochemistry demonstrated a predominantly CD3/CD8-positive T-cell infiltrate with preserved reactive B-cell follicles, while EBV, fungal, and acid-fast bacillus studies were negative. The overall morphologic and immunophenotypic findings favored KFD over lymphoma.

A major diagnostic challenge was distinguishing KFD from lupus lymphadenitis because the two conditions share considerable clinical and histopathological overlap [1,3,6]. Although similar findings may be observed in both conditions, the presence of neutrophils, hematoxylin bodies, and the Azzopardi phenomenon has been described as favoring lupus lymphadenitis over KFD [6]. In our patient, the absence of a significant neutrophilic infiltrate, together with the overall morphologic and immunophenotypic pattern described above, favored KFD, while lupus lymphadenitis remained an important differential diagnosis [1,6]. Secondary HLH was also considered based on the clinical manifestations and laboratory findings; however, an HScore of 92 corresponded to an estimated probability of approximately 1%, making HLH unlikely [7].

Although KFD is generally self-limiting, corticosteroids and other immunomodulatory therapies may be considered in severe or SLE-associated disease [1,2,6]. Hydroxychloroquine has also been reported as a treatment for KFD [8]. In our patient, corticosteroid therapy was escalated and hydroxychloroquine was continued, with subsequent resolution of fever and sustained clinical improvement. This case underscores the diagnostic challenge of KFD in patients with SLE, particularly in the presence of atypical lymphadenopathy, and emphasizes the importance of histopathologic evaluation in distinguishing KFD from lupus lymphadenitis and other mimics.

## Conclusions

This case highlights the close association between KFD and SLE and the diagnostic complexity arising from their overlapping clinical manifestations. The coexistence of these conditions should be considered in patients with SLE who develop persistent fever and prominent lymphadenopathy, particularly when the presentation is atypical for their usual disease course. KFD may closely resemble an active lupus flare or a lymphoproliferative disorder, making a broad differential diagnosis essential. Recognition of this association may facilitate timely diagnosis and appropriate clinical decision-making.
